# Optimization of the Double-Expansion Film-Cooling Hole Using CFD

**DOI:** 10.3390/e25030410

**Published:** 2023-02-24

**Authors:** Zhen Zhang, Tianyu Hu, Xinrong Su, Xin Yuan

**Affiliations:** Department of Energy and Power Engineering, Tsinghua University, Haidian Distrct, Beijing 100084, China

**Keywords:** film cooling, double-expansion hole, numerical optimization, genetic algorithm, aerodynamic loss

## Abstract

Film cooling is a major cooling technique used in modern gas turbines and air engines. The geometry of film-cooling holes is the fundamental aspect affecting the cooling performance. In this paper, a new cooling configuration called the double-expansion film-cooling hole has been put forward, which yields better performance than the widely used shaped holes and is easy to manufacture. The double-expansion holes at inclination angles of $\alpha =30^{\circ }$, $45^{\circ }$, and $60^{\circ }$ are optimized using the genetic algorithm and the Kriging surrogate model, which is trained by CFD data randomly sampled using the Latin hypercube method. The numerically optimized double-expansion holes at different inclination angles were experimentally evaluated and compared with the optimized single-expansion laid-back fan-shaped holes, and the optimized double-expansion hole at $\alpha =30^{\circ }$ was manually modified based on experiment results. Compared with the optimal single-expansion holes, the area-averaged cooling effectiveness of the double-expansion holes was increased by 34.5% at $\alpha =30^{\circ }$, by 27.8% at $\alpha =45^{\circ }$, and basically the same at $\alpha =60^{\circ }$, showing the benefit of the double-expansion concept. The loss mechanism of film cooling was also analyzed in the perspective of the entropy generation rate, showing the optimal double-expansion holes have 21% less loss compared to a baseline narrow single-expansion hole. It was also found that CFD sometimes predicts a different trend from the experiment in optimization, and the experimental validation is necessary.

## 1. Introduction

Film cooling [1,2] is one of the most important cooling techniques used in modern gas turbines and air engines. The basic idea behind film cooling is to deliver coolant from discrete cooling holes to form a thin film of cold fluid that protects the metal wall from being burnt by the mainstream with a temperature higher than the metal melting point. One of the key aspects that affect the cooling performance is the geometry of the cooling holes. The cooling jet slows down when it flows through the expansion part of a shaped hole. The reduced momentum flux makes the cooling jet more likely to cover and protect the surface instead of separating from it. The flow mechanism within the cooling hole is very complicated due to the non-linearity of the Navier–Stokes equation. Therefore, the design of the hole geometry keeps an important and open question in the turbomachinery community.

There are many studies on the shape of film-cooling holes. The effect of the hole shape on the cooling performance was reviewed by Ekkad and Han [3] and Zhang et al. [4]. Basic hole shapes include slots and cylindrical holes. For the renovation of the cylindrical hole, the laid-back hole, the fan-shaped hole, and the laid-back fan-shaped hole were put forward, all of which had an expansion part in different directions. The basic idea of these shaped holes was to reduce the momentum flux of the cooling jet in the expansion part and to make it not separate from the wall. In addition, new configurations such as the converging slot [5], the trench hole [6], the double-jet hole [7], and the anti-vortex hole [8] were explored to further enhance the cooling performance. These new configurations aimed at controlling the fluid dynamics to weaken or break vortex structures downstream the coolant ejection; however, many types of newly developed holes are difficult to manufacture. Fluid dynamics are hard to control, especially in varying flow conditions. Currently the shaped holes are the mainstream and are widely used in real engines. Therefore, exploring the further improvement of the shaped holes is of great significance.

There are various methods used for shape optimization. Generally, the optimization algorithms can be divided into gradient-based and global categories. The adjoint method [9] is the most popular way of gradient-based optimization in CFD, and it has been used in hole optimization [10,11]. The gradient-based optimization can deal with complicated shapes, but it starts with a baseline and is susceptible to local minimum. The global optimization usually adopts the genetic algorithm to search in a huge design space. This strategy has also been used in hole shape optimization [12,13]. The evaluation in the genetic algorithm can be achieved by empirical correlations, surrogate models, and pure CFD simulations. The parameter optimization of shaped film cooling holes involves a small number of design variables with large ranges, so the global optimization strategy is more fitted.

In this paper, a new cooling configuration, named the double-expansion hole, is put forward; it has an extra expansion part compared with the conventional single-expansion laid-back fan-shaped hole. The geometric parameters of the double-expansion hole were then optimized using the genetic algorithm and the Kriging surrogate model [14,15]. The Kriging model was trained using CFD data, which were sampled using the Latin hypercube method [16] within the design space. Three optimal double-expansion holes at different inclination angles were obtained and experimentally compared with the optimized laid-back fan-shaped holes. The area-averaged cooling effectiveness was increased by 34.5% at $\alpha =30^{\circ }$, by 27.8% at $\alpha =45^{\circ }$, and basically the same at $\alpha =60^{\circ }$. CFD can predict the right trend sometimes, and the CFD-based optimization improves the performance remarkably. However, experiment validation is always necessary, because sometimes the numerical and experimental trends are different, which is mainly caused by the insufficient turbulence mixing of the existing RANS models [17].

## 2. Design Concept of the Double-Expansion Hole

**Figure 1:**
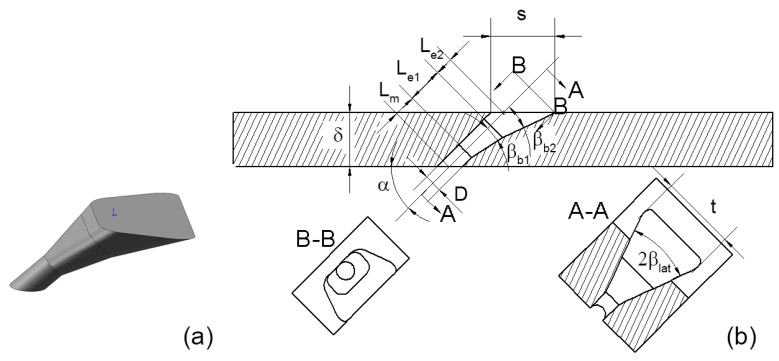
Sketch of the double-expansion hole. (**a**) 3D view. (**b**) Parametric drawing.

Figure 1 shows the sketch of the double-expansion hole. There are two expansion parts, whose lateral-expansion angles $\beta_{lat}$ are the same while laid-back angles $\beta_{b}$ are different. The lengths of the two expansion parts are $L_{e1}$ and $L_{e2}$, respectively, and the length of the metering part is $L_{m}$. Given the thickness of the wall δ, the inclination angle α determines the total length of the hole $L=L_{m}+L_{e1}+L_{e2}$. The diameter of the metering part is D=1 mm. *t* and *s* are the width and length of the hole exit area, indicating the range on the metal surface drilled by the cooling hole. The major difference between the double-expansion hole and the conventional single-expansion laid-back fan-shaped hole is the existence of a second expansion part that further expands in the laid-back direction, i.e. $\beta_{b2}>\beta_{b1}$. The basic idea of this double-expansion hole is based on the trade-off between the cooling performance and the structural strength of the drilled blade. The jet flow angle and the coolant coverage are affected by the lateral-expansion angle $\beta_{lat}$ and the final laid-back angle $\beta_{b2}$. At the same time, the area drilled by the hole, which is represented by *t* and *s*, is also determined by these expansion angles. Therefore, a two-step expansion is added to reduce *s* and *t* while maintaining $\beta_{lat}$ and $\beta_{b2}$, hopefully increasing the cooling effectiveness while satisfying structural constraints. In addition, reducing the $\beta_{lat}$ in the first expansion part is shown to remarkably reduce cooling performance, so only the expansion in the laid-back direction is different for the two expansion parts. Basically, this two-step expansion design adds an extra design parameter and thus extends the design space of the laid-back fan-shaped hole. Therefore, parameter optimization of the double-expansion hole is likely to achieve higher cooling performance under the constraints of *s* and *t*. The double-expansion hole is easy to manufacture, compared to many new holes with complicated geometry, which makes current design concept of high serviceability.

When the inclination angle changes, it is natural to keep the wall thickness δ constant. Three inclination angles of $\alpha =30^{\circ },\;45^{\circ }$ and $60^{\circ }$ are considered, and the wall thickness of t=3D is used. The lateral expansion angle $\beta_{lat}$ and expansion lengths $L_{e1}$ and $L_{e2}$ are also important parameters affecting the final cooling performance, so they are considered in the optimization process.

## 3. Optimization Process

The double-expansion holes at inclination angles of $\alpha =30^{\circ },\;45^{\circ }$, and $60^{\circ }$ are optimized. Table 1 shows the design spaces for all three holes. There are five design variables, allowing the length and expansion angles of the two expansion parts to vary freely. The total length of the hole is determined by the inclination angle given the wall thickness and the hole diameter, and the range of the expansion length $L_{e1}+L_{e2}$ is set accordingly. It has been shown that a large expansion angle can increase the cooling performance, so design spaces of the lateral and laid-back expansion angles are larger than traditional ones [18].

The optimization objective is calculated based on the area-averaged adiabatic film cooling effectiveness η at 5<x/D<30. Four blowing ratios $M=\frac{\rho_{c}u_{c}}{\rho_{m}u_{m}}=1,\;1.5,\;2,$ and 2.5 are considered, and the objective function is $F=(\eta_{1}+\eta_{1.5}+\eta_{2}+\eta_{2.5})/4$. The adiabatic film cooling effectiveness η is defined as

(1) $$\eta =\frac{T_{m}-T_{w}}{T_{m}-T_{c}},$$

where $T_{m}$ is the mainstream temperature, $T_{c}$ is the coolant temperature, and $T_{w}$ is the wall temperature at the adiabatic condition. η indicates how well the coolant covers the metal surface without the heat flux into the wall.

The constraints of this optimization problem are

(2) $$\frac{s}{P}<1,\;\frac{t}{P}<\frac{2}{3},$$

where P=6D is the pitch-wise hole distance in a row.

The optimization algorithm is the genetic algorithm, and a python package named “gaft” is used to implement the genetic algorithm. In genetic algorithm an evaluator of the objective function is needed. The Kriging model implemented in the python package “SMT” [19] is trained to predict the cooling performance given a set of geometric parameters. The automatic formation of the hole shape is achieved based on the open cascade [20].

Randomly sampled design vectors by the Latin hypercube method are used to train the Kriging model. Each design vector contains five variables shown in Table 1, and they are normalized to 0–1 based on maximum and minimum values. Sample amounts for $\alpha =30^{\circ }$, $45^{\circ }$, and $60^{\circ }$ are all 70.

**Figure 2:**
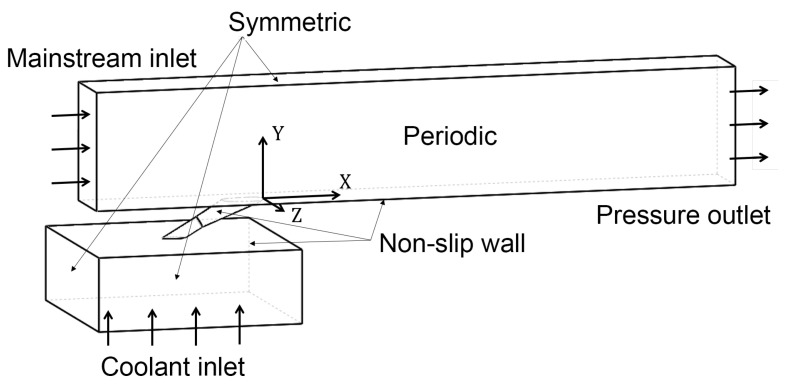
The calculation domain of the single hole film cooling.

The CFD method is used to calculate these samples. Figure 2 shows the calculation domain of the single hole film cooling problem. The domain contains a mainstream channel, a single hole, and a pressure plenum. Two sides of the mainstream channel are periodic, while the channel upper wall and side walls of the plenum are symmetric. Non-slip walls are set to adiabatic. When the blowing ratio changes, the mass flow rate of the coolant inlet is adjusted while the mainstream velocity is fixed. The coordinate origin is located at the middle of the downstream edge of the hole exit. The calculations are performed with the pressure-based solver in ANSYS Fluent. The mainstream temperature is set 300 K, while the coolant temperature is set 200 K, leading to a density ratio of DR=1.5. The mainstream velocity is set 25 m/s. For each design vector, four simulations of different blowing ratios are performed, and this process is automatically organized by a combination of Python and script files.

**Figure 3:**
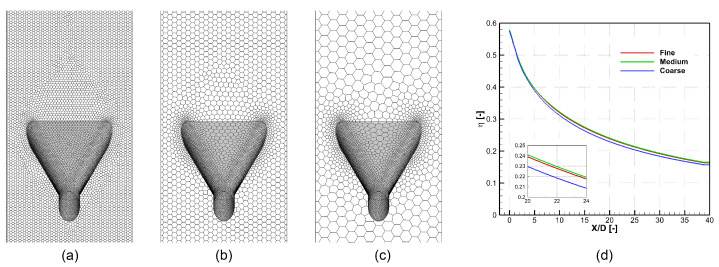
Mesh used for calculation. (**a**) Fine mesh. (**b**) Medium mesh. (**c**) Coarse mesh. (**d**) Comparison of laterally averaged η calculated by different meshes.

Figure 3 compares three sets of meshes of the 45–double hole. All three sets of unstructured meshes are generated by the meshing tool of ANSYS Fluent. Table 2 shows details of these meshes. All three sets of meshes share the same boundary treatment, where the thickness of the first layer of mesh on the wall is $1.0\times 10^{-6}\;$ m, and $y^{+}<0.6$ holds everywhere. Meshes out of the boundary later are controlled by the maximum mesh size shown in Table 2. The difference between results of the medium and the fine meshes is less than 1%, so the size criterion of the medium mesh is used in the rest of the paper.

**Figure 4:**
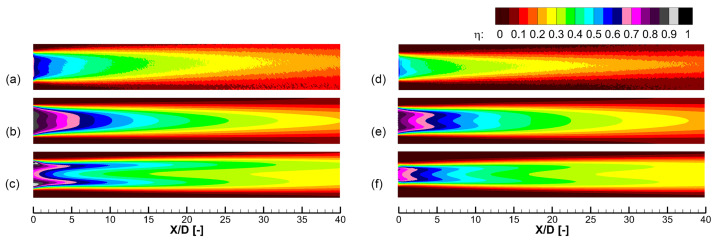
Distributions of the cooling effectiveness predicted by experiment and two turbulence models. Two holes labeled 30–9–14 and 45–9–16 in Table 5 are shown, M=2.0. (**a**) Experiment, 30–9–14 hole. (**b**) Realizable *k*-*ε* model, 30–9–14 hole. (**c**) SST *k*-*ω* model, 30–9–14 hole. (**d**) Experiment, 45–9–16 hole. (**e**) Realizable *k*-*ε* model, 45–9–16 hole. (**f**) SST *k*-*ω* model, 45–9–16 hole.

Figure 4 shows cooling effectiveness distributions predicted by experiment and different turbulence models, including the realizable *k*-*ε* model and the SST *k*-*ω* model. Results of two cooling holes labeled 30–9–14 and 45–9–16 are shown. In the prediction of the 30–9–14 hole, the SST *k*-*ω* model shows a distinct double-peak pattern that is different from the experiment. As for the 45–9–16 hole, the SST *k*-*ω* model predicts separation within the cooling hole, which leads to a narrow jet width, as shown in (f) near X/D=0. This separation is non-physics and remarkably reduces the laterally averaged cooling effectiveness. Compared with the SST *k*-*ω* model, the realizable *k*-*ε* model better predicts the distribution of the cooling effectiveness. However, both turbulence models predict much higher peak value near the hole exit than the experiment, showing the insufficient turbulence mixing of the RANS method in the jet-in-cross-flow problem.

**Figure 5:**
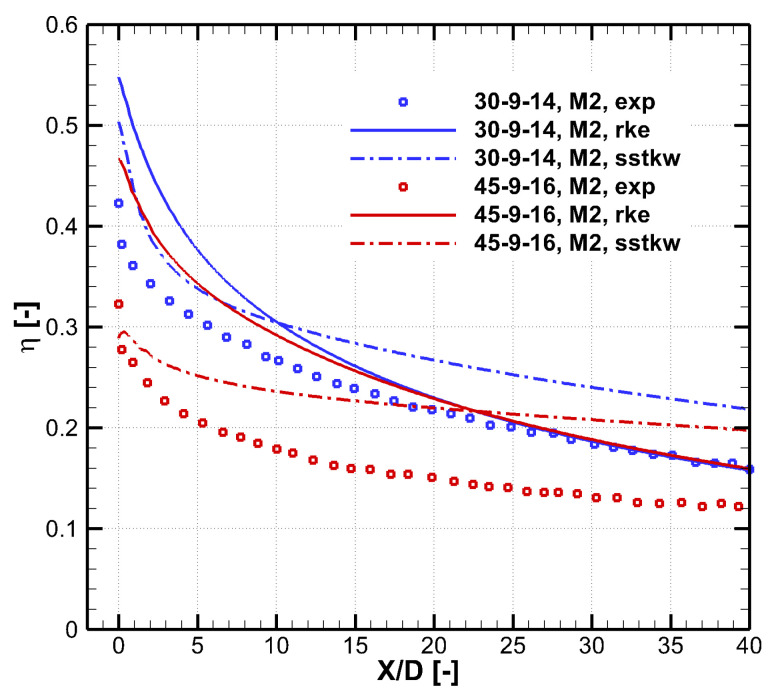
Laterally averaged cooling effectiveness of the 30–9–14 and 45–9–16 holes predicted by the experiment and two turbulence models.

Figure 5 shows the laterally averaged effectiveness of the results shown in Figure 4. Errors of the two turbulence models are both pronounced. The RANS method is know to under-predict the mixing between the cooling jet and the mainstream, so it over-predicts the cooling effectiveness for these large expansion holes. In the experiment, the cooling jet undergoes intense turbulent mixing with the mainstream, and the cooling effectiveness reduces quickly in the flow direction. For narrow expansion holes such as the 30–7–7 hole [18], comparison by the authors [21] shows that the realizable *k*-*ε* model predicts well the laterally averaged cooling effectiveness. Table 3 shows the area averaged cooling effectiveness of the results shown in Figure 5. For the 30–9–14 hole, the realizable *k*-*ε* model predicts better. For the 45–9–16 hole, as shown in Figure 4, the SST *k*-*ω* predicts the non-physical separation within the hole and thus a narrow coolant coverage. Therefore, the realizable *k*-*ε* turbulence model is chosen. However, it should be emphasized that CFD provides a partially reliable trend and the result of the numerical optimization must be evaluated experimentally considering the distinct deviation of the RANS method.

**Figure 6:**
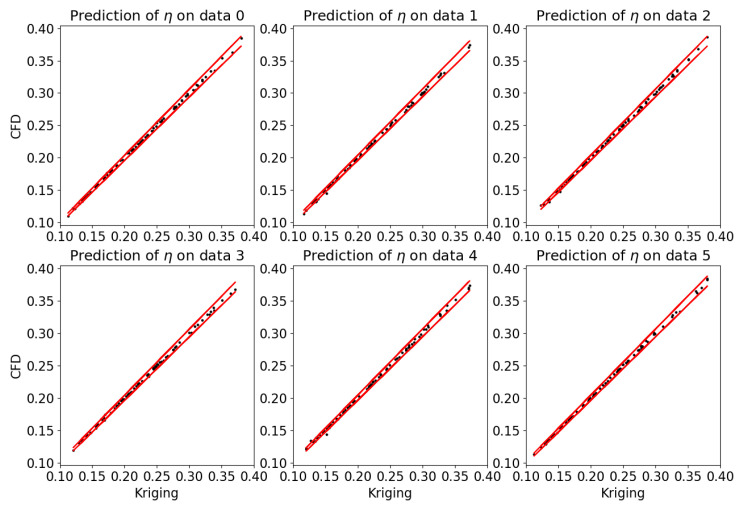
Prediction of the Kriging model trained at $\alpha =30^{\circ }$. The 70 samples are randomly divided into two parts, 70% for training and 30% for test. Six of such tests are performed, and the red line indicates the 2% error.

Three Kriging models are trained using CFD data of $\alpha =30^{\circ }$, $45^{\circ }$, and $60^{\circ }$. Figure 6 shows the prediction at $\alpha =30^{\circ }$. It is shown the model error are basically under 2%. Considering this prediction is an interpolation problem in the design space, the Kriging model trained by all of the samples will predict more accurately, which is sufficient for the optimization.

**Figure 7:**
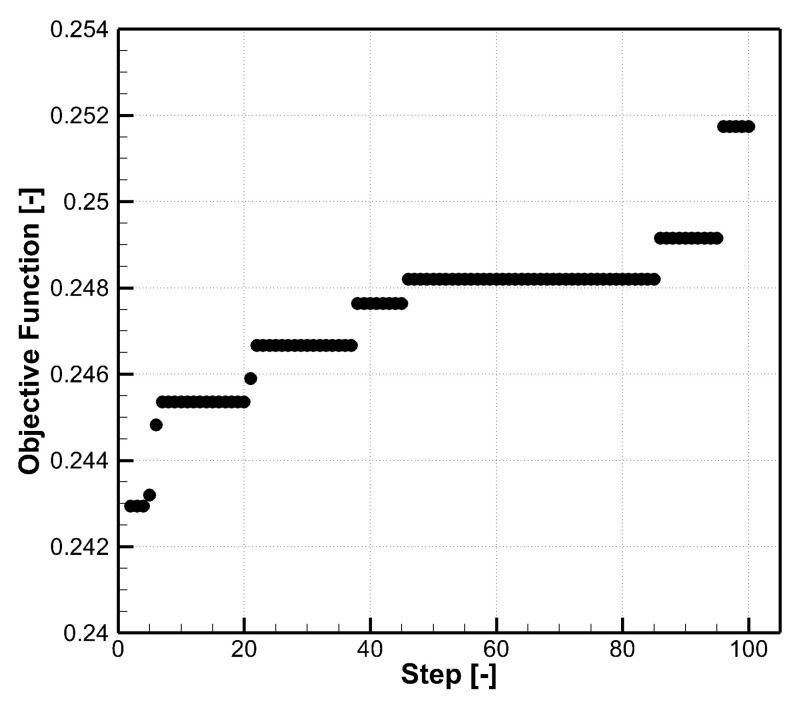
An optimization history at $\alpha =30^{\circ }$.

Figure 7 shows one of the optimization histories at $\alpha =30^{\circ }$. The population size of the genetic algorithm is 100, which means there are 100 design vectors updated by crossover, mutation, and selection in the genetic algorithm. In each step, the largest objective function of these 100 design vectors is plotted, and there are 100 steps in total. For each inclination angles, 50 optimizations starting from different random initial populations are performed to get the final optimal design. This treatment is helpful to find the global maximum, and it is found that results from all 50 runs are similar.

**Figure 8:**
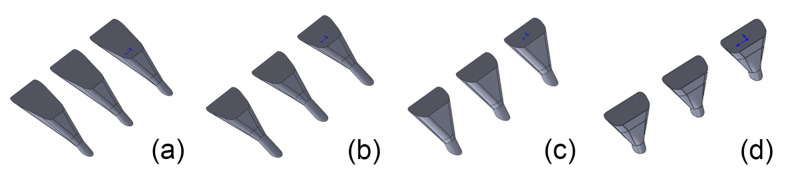
Optimal designs of the double-expansion holes. (**a**) 30–double–1. (**b**) 30–double–2. (**c**) 45–double. (**d**) 60–double.

Table 4 and Figure 8 show the resultant optimal designs of the double-expansion holes. Note that there are two columns related to $\alpha =30^{\circ }$. The first column is the direct output of the numerical optimization. However, the experiment evaluation shows that it is worse than the optimized laid-back fan-shaped hole 30–9–14. Therefore, the design of $\alpha =30^{\circ }$ is manually modified after several iterations of redesign and experimental evaluation. The optimal results of $\alpha =45^{\circ }$ and $60^{\circ }$ show good performance compared with the corresponding shaped holes and thus need no further modification.

## 4. Optimization Results and Experimental Validation

In this section, the optimized double-expansion holes are experimentally compared with the reference laid-back fan-shaped holes that were optimized and validated previously. Table 5 shows the geometric parameters of the reference laid-back fan-shaped holes. Film-cooling holes are named in the form of “α-$\beta_{b}$-$\beta_{lat}$”. They are very close to the real optimal design and are used as reference in the evaluation of the optimized double-expansion hole. There is only one expansion part for these laid-back fan-shaped holes, whose length is $L_{e}$. The laid-back angle and the lateral expansion angle are $\beta_{b}$ and $\beta_{lat}$, respectively.

**Table 5:** Optimal laid-back fan-shaped angles for reference.

Parameter	30–9–14	45–9–16	60–9–25
α	$30^{\circ }$	$45^{\circ }$	$60^{\circ }$
L/D	6.0	4.24	3.46
$L_{e}/D$	3.5	3.5	2.9
$\beta_{lat}$	$14.0^{\circ }$	$16.0^{\circ }$	$25.0^{\circ }$
$\beta_{b}$	$9.0^{\circ }$	$9.0^{\circ }$	$9.0^{\circ }$
s/P	0.65	0.41	0.30
t/P	0.59	0.57	0.66

**Figure 9:**
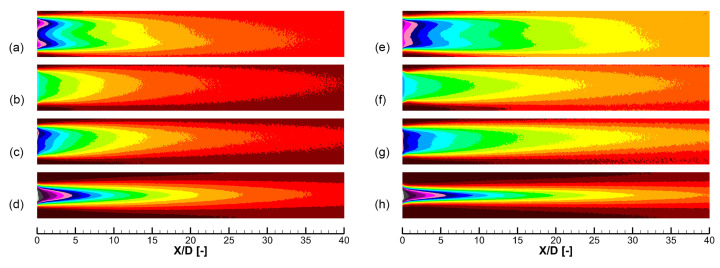
Experiment of 2D cooling effectiveness distributions of different holes at $\alpha =30^{\circ }$. (**a**) 30–double–2, $\beta_{lat}=14.0^{\circ }$, M=1.0. (**b**) 30–double–1, $\beta_{lat}=10.5^{\circ }$, M=1.0. (**c**) 30–9–14, M=1.0. (**d**) 30–7–7 [18], M=1.0. (**e**) 30–double–2, M=2.0. (**f**) 30–double–1, M=2.0. (**g**) 30–9–14, M=2.0. (**h**) 30–7–7, M=2.0.

**Figure 10:**
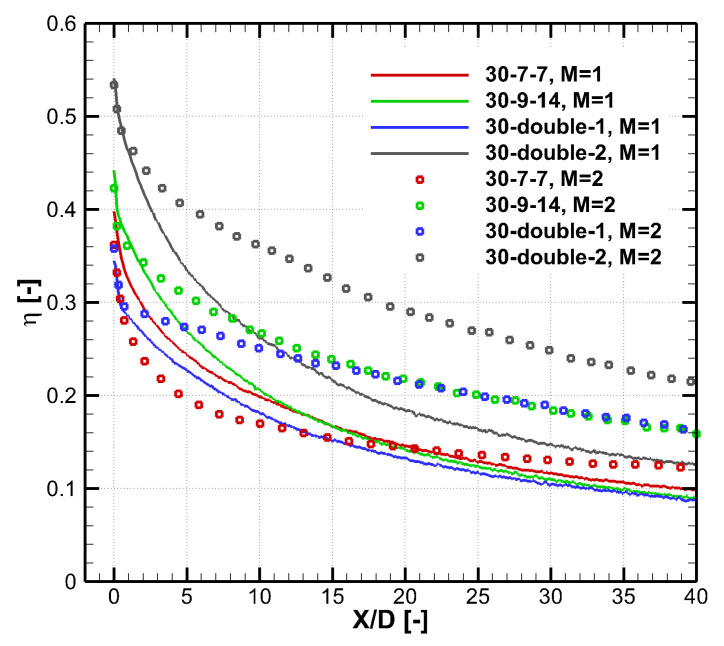
Laterally averaged cooling effectiveness of different holes of $\alpha =30^{\circ }$ at different blowing ratios, experiment measurement.

Figure 9 shows the 2D distribution of the cooling effectiveness of different holes at $\alpha =30^{\circ }$. These results are measured by the pressure-sensitive painting (PSP) method [22], which is illustrated by Chen et al. [23]. Four cooling configurations are compared, namely a baseline laid-back fan-shaped hole 30–7–7 proposed by Schroeder and Thole [18], the optimal laid-back fan-shaped hole 30–9–14 in Table 5, and two double-expansion holes in Table 4. Results of two blowing ratios of M=1.0 and 2.0 are shown. In addition, the corresponding laterally averaged cooling effectiveness are shown in Figure 10.

There are two aspects to evaluate the coolant coverage, i.e., the jet width and the decay speed. Compared with the 30–7–7 hole whose t/P=0.35, all three optimized holes have larger *t* and wider jet width, which can be seen in Figure 9. Given the t/P constraint satisfied, wider hole exit generally leads to better cooling performance. There are two ways to increase the hole exit width *t*, namely increasing the lateral expansion angle $\beta_{lat}$ and increasing the laid-back angle $\beta_{b}$. The numerical optimization selects the second way. The optimization outputs the 30–double–1 hole with a relatively small lateral expansion angle of $\beta_{lat}=10.5^{\circ }$ and a relatively large laid-back angle of $\beta_{b2}=13.0^{\circ }$. Based on the experimental results, the double-expansion hole is manually modified. In 30–double–2 hole, $\beta_{lat}$ is increased, and $\beta_{b1}$ and $\beta_{b2}$ are reduced. As shown in Figure 9 and Figure 10, the 30–double–2 hole over-performs all other holes. When the blowing ratio is M=2.0, the 5<X/D<30 area-averaged η of the 30–double–2 hole is larger than the 30–9–14 hole by 34.5%. This improvement is partly because of the larger design space of the double-expansion holes than that of the traditional laid-back fan-shaped hole.

**Figure 11:**
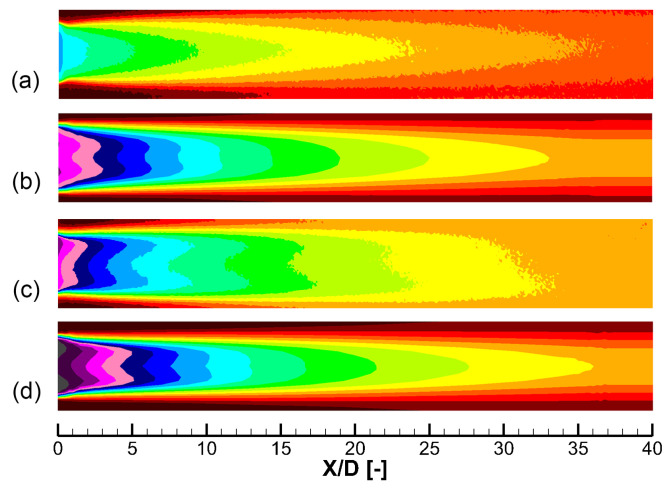
Comparison between experiment and CFD results of cooling effectiveness distributions of two double-expansion holes at $\alpha =30^{\circ }$. (**a**) Experiment of the 30–double–1, $\beta_{lat}=10.5^{\circ }$, M=2.0. (**b**) Calculation of the 30–double–1, M=2.0 (**c**) Experiment of the 30–double–2, $\beta_{lat}=14.0^{\circ }$, M=2.0. (**d**) Calculation of the 30–double–2, M=2.0.

**Figure 12:**
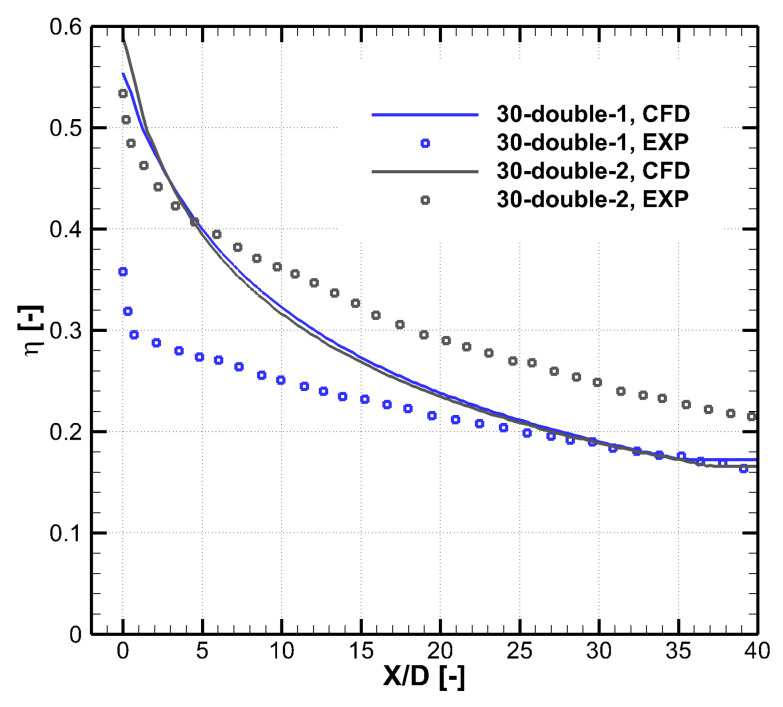
Comparison between experiment and CFD results of laterally averaged cooling effectiveness of two double-expansion holes at $\alpha =30^{\circ }$.

Figure 11 and Figure 12 show the comparison between experimental and numerical results of cooling effectiveness of two double-expansion holes at $\alpha =30^{\circ }$. Although the performance of the 30–double–1 hole predicted by CFD is slightly better than that of the 30–double–2 hole, the experiment shows that the 30–double–1 hole has a larger decay speed of coolant coverage, and the initial value of cooling effectiveness is highly reduced due to turbulence mixing in the long hole exit region (s/P=0.99). There is a large difference between numerical and experimental trends at $\alpha =30^{\circ }$, which introduces difficulty into the numerical optimization. Therefore, accuracy of CFD, especially of turbulence models, needs to further improve to help numerical optimization better guide the engineering design.

**Figure 13:**
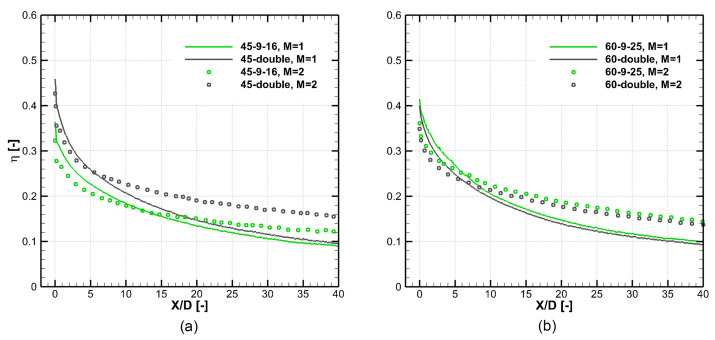
Laterally averaged cooling effectiveness of optimal holes at different blowing ratios, experiment measurement. (**a**) Results of $\alpha =45^{\circ }$. (**b**) Results of $\alpha =60^{\circ }$.

Figure 13 shows the PSP-measured laterally averaged cooling effectiveness of optimal laid-back fan-shaped and double-expansion holes at $\alpha =45^{\circ }$ and $60^{\circ }$. The numerical and experimental trends are similar at these two inclination angles, so the numerical optimization results are regarded as the optimal designs. When the inclination angle is $45^{\circ }$, the optimal 45–double hole has a large lateral expansion angle of $\beta_{lat}=22.5^{\circ }$ and a small laid-back angle of $\beta_{b2}=3.5^{\circ }$. This leads to a slot-like hole exit, and this configuration is shown to have better cooling performance than the 45–9–16 laid-back fan-shaped hole. When the blowing ratio is M=2.0, the 5<X/D<30 area-averaged η of the 45–double hole is larger than the 45–9–16 hole by 27.8%. When the inclination angle is $60^{\circ }$, the optimal 60–double hole and the 60–9–25 hole have similar geometric parameters and thus have similar cooling performance. When the blowing ratio is M=2.0, the 5<X/D<30 area-averaged η of the 60–double hole is smaller than the 60–9–25 hole by 5.7%. The lateral expansion angle of $\beta_{lat}=25.5^{\circ }$ of the 60–double hole is very large compared with the conventional designs, and the experiment shows good cooling effectiveness and no jet separation within the cooling hole. In addition, 5<X/D<30 area-averaged η of the optimal double-expansion holes at $\alpha =30^{\circ }$, $45^{\circ }$, and $60^{\circ }$ are 0.314, 0.203, and 0.201, respectively. The larger the inclination angle is, the easier it is for the cooling jet to separate from the wall, which is harmful to the thermal protection; therefore, the hole of $\alpha =30^{\circ }$ performs the best. However, it is interesting that the reduction of cooling effectiveness from $\alpha =45^{\circ }$ to $60^{\circ }$ is slight, which is a good feature for cooling design at large inclination angles.

## 5. Loss Property of the Double-Expansion Holes

The interaction between cooling jets and the mainstream is an important source of the aerodynamic loss in modern gas turbines. Compared to the widely used parameters such as pressure loss coefficient [24], entropy based analysis is able to provide detailed information about the source of the irreversibility [25,26] and is especially for case such as the strong mixing process in the film cooling. The entropy generation rate is a good indicator showing in detail the loss distribution. According to the energy conservation and the Gibbs equation, one can derive the following formulation of the entropy generation rate.

(3) $$\rho \frac{DS}{Dt}=\frac{\rho \dot{Q}}{T}-\frac{1}{T}\frac{\partial q_{i}}{\partial x_{i}}+\frac{1}{T}\tau_{ij}\frac{\partial u_{i}}{\partial x_{j}}$$

The viscosity loss can be represented as the dissipation function Φ, which is the source of aerodynamic loss. The dissipation function can be formulated as

(4) $$\frac{\Phi }{T}=\frac{1}{T}\tau_{ij}\frac{\partial u_{i}}{\partial x_{j}},$$

where τ is the stress tensor. The above derivation is based on the laminar flow or DNS data of the turbulent flow. When the RANS data is analyzed, Zhao et al. [26] shows that putting the following effectiveness stress tensor into Equation (4) can represent most part of the viscous entropy generation (>90%),

(5) $$\tau_{ij}=\rho \left(\nu_{t}+\nu_{l}\right)\left(\frac{\partial u_{i}}{\partial x_{j}}+\frac{\partial u_{j}}{\partial x_{i}}\right)=\rho \nu_{eff}\left(\frac{\partial u_{i}}{\partial x_{j}}+\frac{\partial u_{j}}{\partial x_{i}}\right).$$

**Figure 14:**
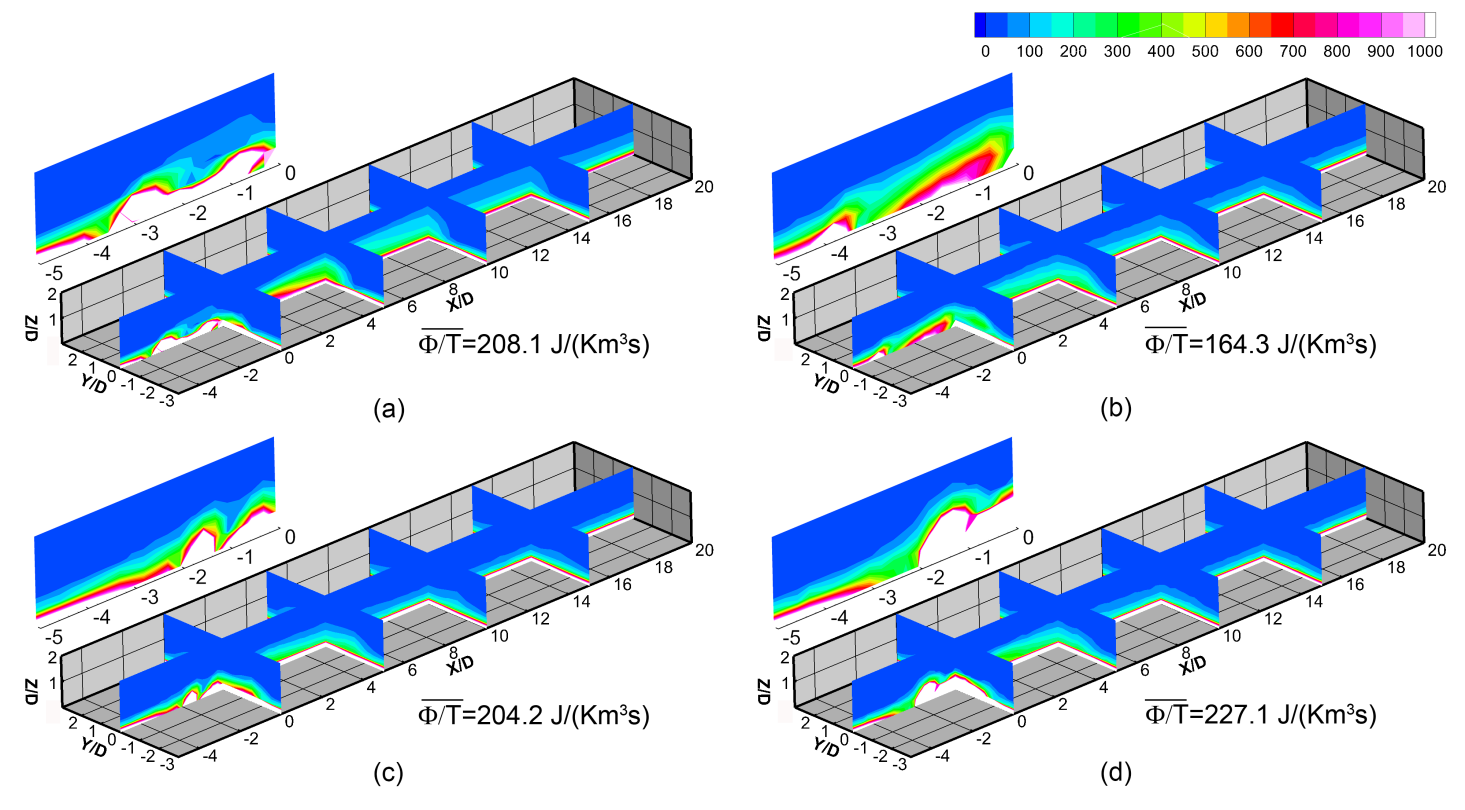
Distribution of the dissipation function Φ/T of different holes, calculated by CFD, M=2.0. The volume-averaged dissipation in this region is also calculated to show the overall loss level. (**a**) 30–7–7. (**b**) 30–double–2. (**c**) 45–double. (**d**) 60–double.

The aerodynamic loss of the double-expansion holes is analyzed. Figure 14 shows distributions of the dissipation function of the baseline 30–7–7 hole ($\alpha =30^{\circ }$) and three optimal double-expansion holes at blowing ratio of M=2.0. The region of −5<X/D<20, −3<Y/D<3, and 0<Z/D<2 is shown, where the major dissipation loss occurs. The averaged dissipation function of these holes are calculated to show the overall level of the aerodynamic loss. The loss mainly occurs near the hole exit and within the boundary layer. It is hard to separate the loss due to jet mixing and that in boundary layer. However, the difference among these configurations shows the effect of jet mixing. Comparison among the double-expansion holes shows that the loss level increases with the inclination angle. It is because the inclination angle increases the velocity difference between the mainstream and the jet at the given blowing ratio. What is more, the comparison between the 30–7–7 hole the 30–double2 hole shows the optimization design also reduces the aerodynamic loss by 21%, because the larger hole expansion leads to lower jet momentum flux and thus lower velocity difference between the jet and the mainstream. It is a good feature for the optimal double-expansion hole to increase cooling performance while reducing aerodynamic loss.

## 6. Discussion and Conclusions

The concept of the double-expansion film-cooling hole is an extension of the conventional single-expansion laid-back fan-shaped cooling hole. The double expansion in the laid-back direction introduces more flexibility to finely control the final jet angle $\beta_{b2}$ and the jet width t/P. Combined with a parameter optimization, the double-expansion design can further increase the cooling performance. The double-expansion hole is easily manufactured using traditional method such as electrical discharge machining.

The optimization of the double-expansion holes at three inclination angles is performed based on CFD results. The ranges of the geometric parameters are designed based on engineering experience to make the design space large enough to include the potential optimal designs. The objective function in the numerical optimization is the area-averaged cooling effectiveness. The constraints of the optimization problem are the length s/P and width t/P of the hole exit area. The optimization algorithm is the genetic algorithm, which is a global optimization method. The problem here is to search in a design space instead of modifying an existed design, so the global method is more fitted than the gradient-based adjoint method. A Kriging model trained and based on CFD data is used to calculate the fitness in the genetic algorithm. A key point here is the ability of CFD to predict the right trend of the objective function. Comparing experimentally the optimized double-expansion holes with the optimal single-expansion laid-back fan-shaped holes, it is found that CFD is moderately successful. The optimized 45–double hole over-performs the 45–9–16 hole by 27.8%, but the 30–double–1 hole drastically under-performs the 30–9–14 hole. Compared with the 30–9–14 laid-back fan-shaped hole, the 30–double–1 hole makes the jet travel a longer distance within the hole and ejects at an angle more parallel to the wall. CFD wrongly predicts the high cooling effectiveness of the configuration. The insufficient turbulent mixing between the jet and the mainstream predicted by the realizable *k*-*ε* turbulence model is the possible reason for the mismatch trends of CFD and experiment. In experiment the effectiveness reduces quickly in the flow direction due to intensive turbulent mixing, while in the CFD result the cooling effectiveness decays much slowly. A dedicated improvement of the turbulence model accuracy at the considering flow conditions is helpful to further improve the numerical optimization. In this work, the double-expansion design at $\alpha =30^{\circ }$ is manually modified and the 30–double–2 hole overperforms the corresponding 30–9–14 laid-back fan-shaped hole by 34.5%. The shapes of these three double-expansion holes can be used as reference in the cooling design of a modern gas turbine.

The loss analysis based on the entropy generation rate shows that the aerodynamic loss caused by the jet mixing increases with the inclination angle. What is more, the optimized double-expansion hole reduces the mixing loss by 21%, compared with the baseline 30–7–7 holes. This is because the larger expansion reduces the momentum flux of the jet and thus weakens the mixing between the jet and the mainstream. 

## Figures and Tables

**Table 1:** Design spaces for double-expansion holes.

Parameter	$\alpha =30^{\circ }$	$\alpha =45^{\circ }$	$\alpha =60^{\circ }$
L/D ^1^	6.00	4.24	3.46
$(L_{e1}+L_{e2})/D$	2–5	2–3.5	2–3
$L_{e1}/\left(L_{e1}+L_{e2}\right)$	0–1	0–1	0–1
$\beta_{lat}$	$7^{\circ }$–$15^{\circ }$	$10^{\circ }$–25${}^{\circ }$	$10^{\circ }$–$30^{\circ }$
$\beta_{b1}$	$1^{\circ }$–$10^{\circ }$	$1^{\circ }$–10${}^{\circ }$	$2^{\circ }$–$15^{\circ }$
$\beta_{b2}$–$\beta_{b1}$	$1^{\circ }$–$10^{\circ }$	$1^{\circ }$–$10^{\circ }$	$2^{\circ }$–$15^{\circ }$

^1^ Not design variable.

**Table 2:** Mesh independency check of the 45–double hole.

Parameter	Fine Mesh	Medium Mesh	Coarse Mesh
Number of cells	$4.3\times 10^{6}$	$7.4\times 10^{5}$	$2.4\times 10^{5}$
Number of nodes	$2.1\times 10^{7}$	$3.2\times 10^{6}$	$7.4\times 10^{5}$
Maximum mesh size	0.125 D	0.25 D	0.5 D
5<X/D<30 averaged η at M=2.0	0.2661	0.2681	0.2574
Error of η	-	0.75%	3.27%

**Table 3:** 5<X/D<30 area averaged cooling effectiveness at M=2.0.

Parameter	30–9–14 Hole	45–9–16 Hole
Experiment	0.233	0.159
Realizable *k*-*ε*	0.255	0.249
SST *k*-*ω*	0.279	0.225

**Table 4:** Optimal designs of the double-expansion holes.

Parameter	30–Double–1 ^1^	30–Double–2 ^2^	45–Double	60–Double
α	$30^{\circ }$	$30^{\circ }$	$45^{\circ }$	$60^{\circ }$
L/D	6.0	6.0	4.24	3.46
$(L_{e1}+L_{e2})/D$	4.9	4.0	3.4	2.9
$L_{e1}/D$	1.0	1.0	0.4	0.8
$L_{e2}/D$	3.9	3.0	3.0	1.1
$\beta_{lat}$	$10.5^{\circ }$	$14.0^{\circ }$	$22.5^{\circ }$	$25.5^{\circ }$
$\beta_{b1}$	$5.5^{\circ }$	$3.0^{\circ }$	$1.5^{\circ }$	$5.0^{\circ }$
$\beta_{b2}$	$13.0^{\circ }$	$9.0^{\circ }$	$3.5^{\circ }$	$10.0^{\circ }$
s/P	0.99	0.64	0.29	0.27
t/P	0.66	0.63	0.66	0.66

^1^ Direct output of the optimization. ^2^ Manually adjusted after experimental validation.

## Data Availability

The data presented in this study are available in article.

## Nomenclature

α	Inclination angle
$\beta_{b}$	Laid-back expansion angle
$\beta_{lat}$	Lateral expansion angle
δ	Wall thickness
η	Cooling effectiveness
Φ	Dissipation function
*D*	Hole diameter
*F*	Objective function
*L*	Hole length
$L_{e}$	Length of the expansion part
$L_{m}$	Length of the metering part
*M*	Blowing ratio
*P*	Pitch-wise hole distance
*s*	Length of hole exit area
*t*	Width of hole exit area
$T_{c}$	Coolant temperature
$T_{m}$	Mainstream temperature
$T_{w}$	Wall temperature
CFD	Computational fluid dynamics
DNS	Direct numerical simulation
PSP	Pressure sensitive paint
RANS	Reynolds-averaged Navier–Stokes
